# Simplification of Supplemental Nutrition Assistance Program Recertification Processes and Association With Uninterrupted Access to Benefits Among Participants With Young Children

**DOI:** 10.1001/jamanetworkopen.2022.30150

**Published:** 2022-09-06

**Authors:** Erica L. Kenney, Mark J. Soto, Michael Fubini, Ashley Carleton, Matthew Lee, Sara N. Bleich

**Affiliations:** 1 Department of Nutrition, Harvard T.H. Chan School of Public Health, Boston, Massachusetts; 2 Department of Health Policy and Management, Harvard T.H. Chan School of Public Health, Boston, Massachusetts; 3 Massachusetts Department of Transitional Assistance, Boston

## Abstract

**Importance:**

In the Supplemental Nutrition Assistance Program (SNAP), families may temporarily lose benefits for which they are still eligible because of administrative issues. This lapse in benefits, referred to as churning, increases the risk of food insecurity for families, which is linked with poorer health.

**Objectives:**

To examine the rate of churning among SNAP participants with young children and evaluate the association of administrative policy changes with churning risk.

**Design, Setting, and Participants:**

A cross-sectional study of recertifications among 9735 SNAP-participating households with at least 1 child younger than 6 years from May to November 2019 tested whether there were sociodemographic differences in churning risk. An interrupted time series analysis of recertifications among 70 799 households from January 1, 2014, to December 31, 2019, tested whether state-level administrative policy changes were associated with churning risk. Data were analyzed between February and November 2021.

**Exposures:**

Three Massachusetts SNAP administrative policy changes.

**Main Outcomes and Measures:**

Churn episodes, defined as losing SNAP benefits after a deadline for eligibility recertification followed by receiving benefits again within 30 days, measured using state SNAP administrative data.

**Results:**

In 2019, a total of 9752 recertification events occurred among 9735 households (3841 [39.4%] Hispanic, 2138 [21.9%] non-Hispanic Black, and 3533 [36.2%] White) with at least 1 child younger than 6 years participating in SNAP. Of these households, 3984 (40.9%) experienced SNAP churning for a period of 1 to 30 days because of missed recertification forms in any given month. Churning was more common among Hispanic households (by 3.9 percentage points; 95% CI, 1.2-6.6 percentage points) than non-Hispanic White households and more common among households with more than 1 child younger than 6 years (by 5.6 percentage points; 95% CI, 3.0-8.1 percentage points) compared with smaller households. Among households that churned, non-Hispanic White households lost a mean of $10.51 (95% CI, $10.16-$10.86) in benefits per household member, and non-Hispanic Black households lost an additional $1.10 (95% CI, $0.47-$1.74) per household member. On implementation of a task-based, first-available-caseworker model, mean churn episode length was significantly shorter immediately on introduction of the model (−0.30 days per each month after the policy change; 95% CI, −0.52 to −0.07 days) and over time (−0.23 days per each month after the policy change; 95% CI, −0.26 to −0.20 days). At the addition of a second policy change to expand participants’ access to simplified reporting requirements, a decrease was seen in the trend in the probability of a churn episode by −0.74 percentage points per month (95% CI, −0.87 to −0.62 percentage points). A third policy to expand online services for submitting recertification paperwork was not associated with a change in churn risk over time.

**Conclusions and Relevance:**

Churning in SNAP is common among households with young children and is disproportionately experienced by Black and Hispanic households and lower-income households. Administrative changes to simplify caseworkers’ workloads and streamline eligibility reporting for participants may reduce churn.

## Key Points

**Questions:**

How frequently do Supplemental Nutrition Assistance Program (SNAP) participants with young children—who are at higher risk for food insecurity—face disrupted benefits because of administrative issues (known as churning), and are specific policies associated with reduced churning?

**Findings:**

In this cross-sectional study of 70 799 Massachusetts SNAP participants with young children, 40.9% of participants experienced benefit losses that lasted up to 30 days because of administrative reasons, including missed deadlines for submitting recertification forms, completing certification interviews, or providing eligibility verifications. Policies that simplified caseworker assignments and reduced the burden of interim eligibility reporting requirements were associated with significant decreases in churning.

**Meaning:**

The findings of this study suggest that SNAP participants with young children are at high risk of losing benefits when facing recertification deadlines; administrative policies may help reduce interruptions in benefit delivery to households with young children.

## Teaser

This cohort study of households participating in the Supplemental Nutrition Assistance Program (SNAP) examines the prevalence of administrative churning among households with children younger than 6 years and explores the association of administrative policy changes with churning risk.

## Introduction

Food insecurity is a major threat to the health and well-being of low-income children, affecting 14.8% of US households with children.^1^ Experiencing food insecurity is associated with poor academic performance,^2^ low fruit and vegetable intake,^3^ and higher medical expenditures.^4^ The consequences of food insecurity are particularly strong for young children, who are at higher risk of anemia, asthma, and generally poorer health in addition to cognitive and behavioral problems.^5^ Given that early childhood is an important period for development, understanding how to mitigate food insecurity for this population is particularly important.

The Supplemental Nutritional Assistance Program (SNAP), administered by the US Department of Agriculture, helps mitigate food insecurity for households with low incomes (typically <130% of the federal poverty level, although some states provide SNAP for households up to 200% of the federal poverty level). SNAP distributes benefits on electronic benefit transfer cards that can be used to purchase foods (except heated, prepared foods) and nonalcoholic beverages. SNAP provided 39.9 million people with $78.9 billion in benefits in 2020^6^ and reaches 1 in 5 children.^7^ SNAP reduces food insecurity and is associated with better overall health, less psychological distress, better birth outcomes, and reductions in health care costs.^8^

However, SNAP is underused in terms of underenrollment^9^ and problems remaining enrolled. A previous study^10^ found that approximately one-half of former SNAP participants had still been eligible to participate when they left the program. Administrative procedures may play a role. To maintain SNAP benefits, households must submit a recertification form, potentially complete an interview with a case manager, and provide documents that verify their circumstances before the end of their certification period, typically 6 to 24 months after applying.^11^ Problems in meeting these requirements, which can be challenging for households experiencing the stressors that accompany poverty, can result in failures to recertify among still-eligible households.^12^ This recertification failure can result in churning, a phenomenon well studied within Medicaid (which similarly requires regular verifications to recertify), wherein a household exits the program, largely because of missed deadlines or incomplete paperwork for eligibility recertification, only to return to the program shortly afterward.^13,14^

Churning is extremely disruptive for both SNAP recipients and administering agencies, resulting in increased food insecurity for families^15^ and added administrative costs.^16^ It may also disproportionately affect participants with the lowest household incomes, particularly households with children, as well as Black families.^17,18^ However, little is known about the prevalence of churning in SNAP among households with young children, who may be particularly harmed by disruptions in SNAP benefits, as well as whether there are racial and ethnic or socioeconomic disparities in churning among these households. It is also unknown whether changes to SNAP case management and benefit administration processes could reduce churning, as has been found in Medicaid.^13,14^

**Figure 1.**
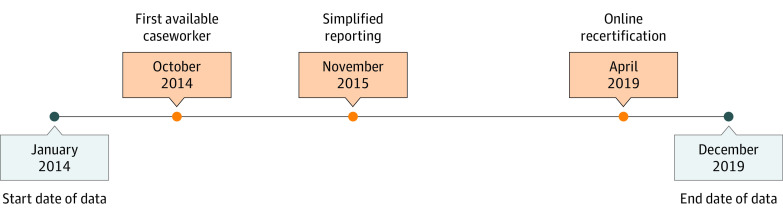
Timeline of Massachusetts Department of Transitional Assistance Administrative Policy Changes to Supplemental Nutrition Assistance Program Recertifications, 2014-2019

The federal government provides states with some flexibility in their administration of SNAP eligibility recertification processes,^11^ producing an opportunity to evaluate whether state innovations in administrative practices could reduce churning. This study evaluates 3 strategies that the Massachusetts Department of Transitional Assistance (DTA), which administers SNAP in Massachusetts, undertook from 2014 to 2019 to reduce SNAP eligibility reporting burdens (Figure 1). These strategies included (1) the task-based model, in which SNAP participants were assigned to the first-available caseworker for all case processes rather than being assigned to a consistent caseworker (beginning October 2014); (2) expanding access to simplified reporting for most SNAP households, in which households were only obligated to report if their income exceeded the threshold and could report changes to household circumstances via a brief report at the halfway point between certification periods rather than being obligated to report changes in circumstance within 10 days of becoming known to the participant (beginning November 2015); and (3) an online recertification option, introducing the ability to complete recertifications online (beginning April 2019). Leveraging 6 years (2014-2019) of SNAP recertification data from the Massachusetts DTA, our study aims were to (1) estimate the current prevalence of and sociodemographic factors associated with administrative churning among households with at least 1 child younger than 6 years and (2) examine the association of the 3 administrative policy changes with churning risk.

## Methods

### Study Design

This cross-sectional study explored sociodemographic factors associated with churning at recertifications among households with young children. An interrupted time series model estimated the association of 3 different administrative policies on SNAP churning risk over time. Participant consent was waived, and the study was deemed exempt by the Harvard T.H. Chan School of Public Health Institutional Review Board because data were deidentified. The study followed the Strengthening the Reporting of Observational Studies in Epidemiology (STROBE) reporting guideline.

### Sample

The data obtained from the Massachusetts DTA described 138 066 deidentified recertification processes for 82 896 households with young children in Massachusetts whose recertification deadlines ranged from January 1, 2014, to December 31, 2019. Data included recertification deadline, actual recertification date, starting and ending dates of benefit delivery, monthly benefit amount, and participant-reported demographic characteristics for the head of household. Recertifications missing race and ethnicity, age, sex, marital status, or town of residence across all recertification forms for that household were excluded (n = 6922 households) (eFigure in the Supplement), as were recertifications from Native American or multiracial households because of relative rarity (n = 665). For the cross-sectional analysis of sociodemographic factors associated with churning, the analytical sample was restricted to recertifications between May and November 2019 (n = 9752 recertification processes among 9735 households). For the interrupted time series analysis of the association of policy changes with churning, data from 6 years (2014-2019) of recertification forms (n = 115 351), representing 70 799 households with young children, were used.

### Outcome Measures

#### Primary Outcome: Churning for Administrative Reasons

In this study, we examined churning due to participants missing deadlines for recertification forms, interviews, or verifications; churning due to administrative errors by the Massachusetts DTA, which does not result in benefit loss to participants, was not evaluated (for more information on recertification procedures and churning, see the eAppendix in the Supplement). Households that failed to renew their eligibility by their recertification deadline but successfully recertified within 30 days of the initial deadline were considered to have churned for that recertification event. This 30-day threshold was used based on a prior analysis^19^ within the Massachusetts DTA, which found that this threshold distinguishes unintentional churners from individuals who may pause participation in SNAP intentionally. Households that successfully renewed their eligibility on time and thus experienced no disruption in benefits were categorized as not churning. Households that failed to renew their eligibility and did not return to SNAP within 30 days were treated as leaving SNAP. Given that churn has also been defined by the US Department of Agriculture using a 120-day window, we also conducted sensitivity analyses using this definition (eTables 1 and 2 in the Supplement).

#### Secondary Outcomes: Length of Churn Episodes, Total Churn Episodes, and Benefits Lost

We calculated the length of each churn episode in days. To estimate whether households had repeated churning episodes, we summed each household’s total churn episodes from 2014 to 2019. We also estimated the value of the total daily SNAP benefits lost per household member because of churn episodes during the study period by multiplying each household’s daily benefit by the number of days the household did not receive SNAP benefits during their churn episode, then dividing by the total number of members of the household.

#### Sociodemographic Covariates

SNAP applicants reported race and ethnicity, total household income, marital status, number of children younger than 6 years, age, and sex of the household member who applied for SNAP benefits. Race and ethnicity were evaluated in this study because prior investigations have found disparate rates of churning by race and ethnicity.

### Statistical Analysis

#### Cross-sectional Analysis of Demographic Factors Associated With Churning

A multivariable linear probability regression model adjusting for all covariates simultaneously estimated sociodemographic factors associated with churn due to missed administrative deadlines using recertification events from May to December 2019 (α < .05). To adjust for potential confounding by economic conditions, we included the local unemployment rate during the month when the recertification form was due. For the secondary outcomes of total churn episodes between January 1, 2014, and December 31, 2019, and benefits lost per churn episode, we constructed generalized linear models with the same covariates. Robust SEs were clustered on town of residence.

#### Interrupted Time Series Analysis of Policy Changes

To analyze whether administrative policy changes by the Massachusetts DTA were associated with changes in the probability and duration of churning, we used an interrupted time series analysis approach^20^ with segmented linear probability regression models that evaluated whether the probability of churning changed each month from before to after each administrative policy change; segmented linear regression models similarly evaluated whether the duration of churning episodes changed.^21^ The models leveraged repeated cross-sectional estimates of the proportion and duration of churning episodes across all recertification forms due or submitted for each month between January 2014 and November 2019.

The models included a term for time (measured in months), which tested whether there was any existing time trend in churning probability or duration per month in the months before the first administrative change. Models also included a pre/post indicator for the time of each administrative change, which tested, for each policy change, whether the probability or duration of churning changed immediately at the time the policy was introduced (October 2014, November 2015, and April 2019). Last, models included a term representing the time (in months) since each administrative change, which tested whether each successive policy change was associated with a change in the time trend of the probability or duration of churning. Models were adjusted for seasonal variation in churn probability by including dummy variables for calendar month. Monthly town-level unemployment rates when a participant’s recertification was submitted or due were included to adjust for local economic changes potentially related to SNAP retention.^22^ Robust SEs were clustered on town of residence. Analyses were conducted between February and November 2021 using Stata software, version 15 (StataCorp LLC). A 2-sided *P* < .05 was considered to be statistically significant.

## Results

The cross-sectional sample (May to November 2019) included 9735 households (3841 [39.4%] Hispanic, 2138 [21.9%] non-Hispanic Black, and 3533 [36.2%] White) with young children due for eligibility recertification, whereas the interrupted time series sample included 70 799 households and 115 351 recertification events (January 2014 to November 2019). The cross-sectional sample had a mean (SD) monthly earned income of $877.01 ($1028.24) and a mean (SD) unearned income of $225.86 ($446.41) (Table 1). Demographic characteristics of the interrupted time series sample were similar.

**Table 1.** Demographic Characteristics of 9735 Households per 9752 Recertification Events Among Households With at Least 1 Child Younger Than 6 Years Participating in SNAP in Massachusetts, May to November 2019^a^

Characteristic	Overall	Successful recertification^b^	Churn <30 d^c^	No return^d^
Overall	9752 (100)	2935 (30.1)	3984 (40.9)	2833 (29.1)
No. of people in a household, mean (SD)	4.24 (1.31)	4.37 (1.33)	4.31 (1.32)	4.01 (1.22)
Earned income, mean (SD), $	877.01 (1028.24)	821.53 (1002.95)	798.58 (961.20)	1044.76 (1121.97)
Unearned income, mean (SD), $	225.86 (446.41)	247.46 (459.12)	222.20 (439.83)	208.63 (441.48)
Monthly SNAP benefit, mean (SD), $	401.78 (194.38)	421.85 (197.88)	425.39 (187.18)	347.79 (190.07)
Length of churn spell, mean (SD), d	NA	NA	10.09 (9.71)	NA
Race and ethnicity				
Hispanic	3841 (39.4)	1174 (40.0)	1652 (41.5)	1015 (35.8)
Non-Hispanic Asian	240 (2.5)	52 (1.8)	99 (2.5)	89 (3.1)
Non-Hispanic Black	2138 (21.9)	611 (20.8)	864 (21.7)	663 (23.4)
Non-Hispanic White	3533 (36.2)	1098 (37.4)	1369 (34.4)	1066 (37.6)
Age group, y				
17-29	4373 (44.8)	1275 (43.4)	1799 (45.2)	1299 (45.9)
30-39	4199 (43.1)	1336 (45.5)	1692 (42.5)	1171 (41.3)
≥40	1180 (12.1)	324 (11.0)	493 (12.4)	363 (12.8)
No. of children <6 y of age				
1	7354 (75.4)	2164 (73.7)	2914 (73.1)	2276 (80.3)
≥2	2398 (24.6)	771 (26.3)	1070 (26.9)	557 (19.7)
Sex				
Female	9111 (93.4)	2783 (94.8)	3758 (94.3)	2570 (90.7)
Male	641 (6.6)	152 (5.2)	226 (5.7)	263 (9.3)

Abbreviations: NA, not applicable; SNAP, Supplemental Nutrition Assistance Program.

^a^ Sample includes eligibility renewal applications from SNAP participant heads of household with at least 1 child younger than 6 years and nonmissing values for race and ethnicity, age, sex, and marital status. Values may not total 100% because of missing values.

^b^ Recertifications occur at the end of a SNAP eligibility period and require proof of income and interview with a case manager by the SNAP participant. Failure to comply with the certification deadline results in termination of SNAP benefits.

^c^ Churn indicates failure to comply with renewal deadline but successfully re-enrolled shortly afterward.

^d^ No return indicates households whose SNAP participation was disrupted between May and November 2019 and did not return to SNAP by December 2019.

From May to November 2019, 3984 households (40.9%) with young children experienced a churn episode of 30 days or less in any given month (Table 1), 2935 (30.1%) successfully renewed their eligibility, and 2833 (29.1%) left the program for 30 or more days. The mean (SD) duration of time that a churning household did not receive SNAP benefits was 10.1 (9.7) days.

### Sociodemographic Factors Associated With Churn for Families With Young Children

In fully adjusted models, compared with non-Hispanic White households (reference group), Hispanic households were 3.9 percentage points (95% CI, 1.2-6.6 percentage points; *P* = .004) more likely to churn when due for recertification (Table 2). Households with more than 1 child younger than 6 years were 5.6 percentage points (95% CI, 3.0-8.1 percentage points; *P* < .001) more likely to experience churn compared with households with only 1 young child. Each additional $1000 in total annual household income was associated with a change in churning risk of −3.2 percentage points (95% CI, −4.1 to −2.3 percentage points; *P* < .001).

**Table 2.** Association of Demographic Characteristics With the Probability of Experiencing a Churn Spell Within 30 Days of Certification Among 9752 Recertification Events Among Households With Young Children in Massachusetts, May to November 2019

Characteristic	Annual recertifications^a^	
	Estimate (95% CI)^b^	*P* value
Age group, y		
18-29 (Reference group)	39.9 (38.2 to 41.6)	<.001
30-39	1.0 (−0.9 to 3.0)	.30
≥40	4.2 (0.5 to 7.9)	.03
Race and ethnicity		
Hispanic	3.9 (1.2 to 6.6)	.004
Non-Hispanic Asian	4.2 (−1.2 to 9.6)	.13
Non-Hispanic Black	1.3 (−1.1 to 3.8)	.28
Non-Hispanic White (reference group)	38.9 (37.4 to 40.4)	<.001
No. of children <6 y of age		
1 (Reference group)	39.5 (38.2 to 40.8)	<.001
≥2	5.6 (3.0 to 8.1)	<.001
Sex		
Female (Reference group)	41.2 (40.1 to 42.3)	<.001
Male	−5.1 (−9.1 to −1.1)	.01
Total household income per $1000 change, $	−3.2 (−4.1 to −2.3)	<.001

Abbreviation: SNAP, Supplemental Nutrition Assistance Program.

^a^ Annual certifications occur at the end of a SNAP eligibility period and require proof of income and interview with a case manager by the SNAP participant. Failure to comply with the certification deadline results in termination of SNAP benefits.

^b^ Estimates are probability (scale, 1-100) of churning for the reference group and differences in the probability of churning from the reference group for each comparison group independent of all other covariates. Models adjust for reported marital status and include fixed effects for month and monthly unadjusted unemployment rate for town of residence. Robust SEs are clustered on participant's town of residence.

### Interrupted Time Series Analysis of Policy Changes

**Figure 2.**
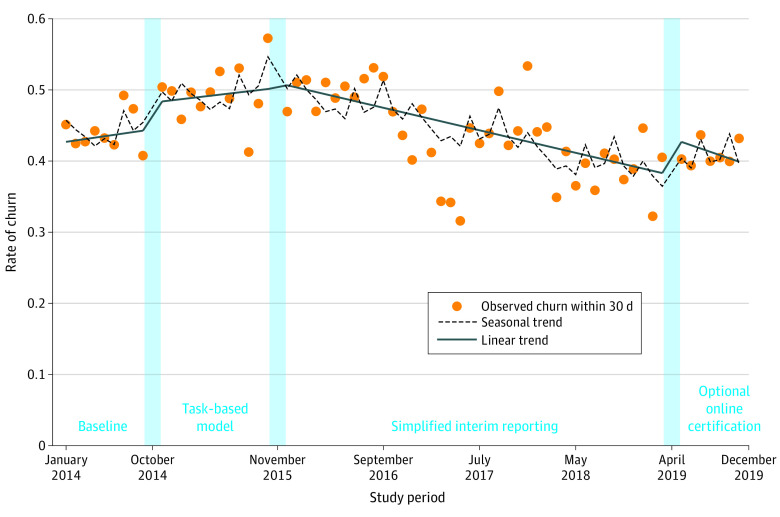
Observed, Seasonal, and Linear Trends in Rate of 30-Day Churn Episodes in Massachusetts Among Households With Children Younger Than 6 Years Participating in the Supplemental Nutrition Assistance Program, 2014-2019 Churning occurs when families temporarily lose benefits for which they are still eligible because of administrative issues.

From January 1 to September 30, 2014, before implementation of the 3 administrative policies, the estimated probability of a household with young children churning within 30 days of recertification was 42.6% (95% CI, 40.9%-44.4%; *P* < .001) and stable from month to month (Figure 2). In October 2014 on implementation of the first policy, the task-based model, there was no significant change in the probability of churning either in the first month of implementation or over time (December 2015). However, churn episode length was significantly shorter, both immediately on introduction of the task-based model (estimate, −0.30 days; 95% CI, −0.52 to −0.07 days) and over time afterward (estimate, −0.23 fewer days per churn episode per month; 95% CI, −0.26 to −0.20 days). At the addition of the second policy change, expansion of simplified reporting, no significant immediate shift in the probability or duration of churning was seen, but from December 2015 to March 2019 while both the task-based model and this new policy were in effect, there was a decrease in the trend in the probability of a churn episode by −0.74 percentage points per month (95% CI, −0.87 to −0.62 percentage points; *P* < .001 for trend), accompanied by a small increase in the duration of churn episodes (0.05 additional churn days per episode per month; 95% CI, 0.05-0.06 days). On May 1, 2019, the third policy, optional online recertification, was associated with an immediate increase in probability of churning of 4.43 percentage points (95% CI, 2.87-5.99 percentage points; *P* < .001), but after this initial increase, churning risk did not significantly change. Policy change associations with churn risk were similar across all demographic groups (eTable 3 in the Supplement).

### Repeated Experiences With SNAP Churning and Dollar Value of Lost Benefits

Across 2014 to 2019, households with at least 1 child younger than 6 years experienced a mean (SD) of 1.99 (1.26) churning episodes, with households that identified as non-Hispanic Black (estimate, 0.13; 95% CI, 0.09-0.18; *P* < .001) or Hispanic (estimate, 0.14; 95% CI, 0.10-0.19; *P* < .001) or that had 2 or more children younger than 6 years (estimate, 0.14; 95% CI, 0.08-0.20; *P* < .001) experiencing more total churn episodes at recertification because of missed administrative deadlines compared with reference groups (Table 3). Higher mean household income was associated with significantly fewer total churn events (estimate, 0.05; 95% CI, 0.03-0.07; *P* < .001). Among households that churned, non-Hispanic White households lost a mean of $10.51 (95% CI, $10.16-$10.86) in benefits per household member, and non-Hispanic Black households lost an additional $1.10 (95% CI, $0.47-$1.74) per household member.

**Table 3.** Number of Churn Episodes per Participant and Total Amount in Benefits Lost per Churn Episode Among Participants Who Experienced at Least One 30-Day Churn Episode, Massachusetts, 2014-2019^a^

Characteristic	Total No. of 30-d churn events^b^		Total amount lost per 30-d churn episode, $^c^		Total amount lost per 30-d churn episode per household member, $^c^	
	Estimate (95% CI)	*P* value	Estimate (95% CI)	*P* value	Estimate (95% CI)	*P* value
Age group, y						
18-29 (Reference group)	2.20 (2.15 to 2.25)	<.001	42.99 (41.28 to 44.71)	<.001	11.33 (10.68 to 11.98)	<.001
30-39	−0.29 (−0.34 to −0.24)	<.001	3.61 (2.22 to 5.01)	<.001	−1.02 (−1.39 to −0.65)	<.001
≥40	−0.68 (−0.85 to −0.51)	<.001	3.63 (1.20 to 6.06)	.003	−1.47 (−2.03 to −0.92)	<.001
Race and ethnicity						
Hispanic	0.14 (0.10 to 0.19)	<.001	−1.31 (−3.49 to 0.86)	.24	−0.12 (−0.76 to 0.52)	.71
Non-Hispanic Asian	0.02 (−0.04 to 0.08)	.51	1.63 (−2.84 to 6.09)	.48	0.76 (−0.62 to 2.15)	.28
Non-Hispanic Black	0.13 (0.09 to 0.18)	<.001	4.14 (0.89 to 7.40)	.01	1.10 (0.47 to 1.74)	<.001
Non-Hispanic White (reference group)	1.94 (1.92 to 1.96)	<.001	44.64 (43.26 to 46.03)	<.001	10.51 (10.16 to 10.86)	<.001
No. of children <6 y of age						
1 (Reference group)	1.97 (1.94 to 2.00)	<.001	42.01 (40.37 to 43.65)	<.001	10.70 (10.18 to 11.22)	<.001
≥2	0.14 (0.08 to 0.20)	<.001	11.77 (9.54 to 13.99)	<.001	−0.00 (−0.39 to 0.38)	.99
Sex						
Female (reference group)	2.04 (2.01 to 2.07)	<.001	44.91 (43.05 to 46.78)	<.001	10.67 (10.10 to 11.24)	<.001
Male	−0.23 (−0.28 to −0.17)	<.001	1.17 (−2.32 to 4.66)	.51	0.51 (−0.19 to 1.21)	.15
Total household income per $1000 change, $	0.05 (0.03 to 0.07)	<.001	2.23 (0.68 to 3.78)	.005	−0.07 (−0.45 to 0.30)	.70

Abbreviation: SNAP, Supplemental Nutrition Assistance Program.

^a^ Sample includes SNAP participant heads of household with at least 1 child younger than 6 years. Models account for reported marital status. Robust SEs were clustered on participant's town of residence.

^b^ Estimates are the mean number of churn episodes during the study period for reference groups or differences from the reference group for each comparison group.

^c^ Estimates are the mean benefits lost per churn episode among households that experience a churn spell lasting 30 days or less for reference groups or differences in lost benefits from the reference group for each comparison group.

## Discussion

This study found that churning, or disruptions to SNAP benefits because of participants missing administrative deadlines for verifying eligibility, was common among households with young children, with 40.9% of recertifications resulting in churning in any given month. Churning was more common than successful recertifications or leaving the program intentionally. Moreover, racial or ethnic and socioeconomic disparities were found in churning, with Hispanic and single-parent households, as well as households with greater numbers of young children and those with lower income, at significantly higher risk. Our results are consistent with prior studies^10,18,23^ of churning in SNAP. Although the duration of these churning episodes seems short (<30 days for most households and resulting in a loss of approximately $44 for a household of 4), these episodes disrupt access to needed benefits for households that are extremely economically disadvantaged. Churning is also costly to the government, with 1 report^16^ estimating that each churn episode costs SNAP administering agencies $80. Churning presents a critical challenge to SNAP’s ability to protect vulnerable families from food insecurity and to operate efficiently.

In addition, our results suggest that administrative policies may reduce churning episodes and the duration of those episodes. The task-based model, in combination with the expansion of simplified reporting, was associated with a steady reduction in the probability of churning at recertification over time. The task-based model alone was also associated with significant reductions in the mean churn episode length, suggesting it helped to minimize the negative impacts of churn even if it did not always fully prevent it. Although the optional online recertification approach was also associated with an initial increase in churn risk, this increase appeared to be leveling off over time as well, although we were not able to evaluate the policy using a longer time frame. Other states that do not already use the task-based model or simplified reporting may want to consider implementing these approaches. States may also want to consider strategies for mitigating possible increases in churn risk during initial rollout phases of new policies.

Although our study demonstrates racial, ethnic, and income inequities in churning as well as evidence for administrative policies that can influence churning rates, our study is unable to identify why these disparities exist or why these policies were associated with different churn rates. Future research is needed to identify why Hispanic, lower-income, and single-parent households were at higher risk of churning and how to target interventions to alleviate churning within these groups. Churning in SNAP, especially among communities of color, may exacerbate long-standing racial and ethnic disparities in food insecurity. In addition, given that churn risk was still quite high even after the policy changes, it is critical to identify further strategies to reduce churning. Investigating how changes to SNAP administrative policies during COVID-19 might have influenced churn risk could be an important next step.

### Strengths and Limitations

Strengths of this study include the use of several years of objective administrative data from the Massachusetts DTA, which avoided reliance on self-report measures of churn and allowed for substantial statistical power. Limitations include the reduced generalizability of the results outside Massachusetts, which has made substantial efforts to reduce administrative burden and improve program administration and has one of the higher rates of participation in SNAP.^24^ Our estimates of the prevalence of churning and of the magnitude of racial or ethnic and other sociodemographic disparities in churning may be underestimates compared with other states. Our interrupted time series analysis could not include a true control group of Massachusetts SNAP participants because all participants were exposed to the policy changes, so we cannot rule out that other factors influenced the observed changes in churning risk. However, to account for underlying time trends or external economic events, we adjusted for baseline trends in churning and community-level, time-varying economic conditions. A final limitation is that we only evaluated churning at recertification events because of missed administrative deadlines, although churning can occur for other reasons (eg, errors on the part of the administering agency) and at other times, such as the interim reports. However, for interim reports, which we could not evaluate because of changes in reporting requirements concurrent to this study (which changed the demographic makeup of those completing interim reports, making the study population inconsistent), exploratory analyses found similar results in terms of both sociodemographic disparities and policy influences.

## Conclusions

This cross-sectional study found that SNAP churning because of missed administrative deadlines was associated with disrupted food assistance benefits for 40.9% of participating households with young children, with disproportionately higher impacts for Hispanic and lower-income households. Administrative approaches that streamline the recertification process for both caseworkers and participants could reduce churning risk. Targeted efforts toward groups with higher risk of churn, as identified in this project, could further address the problem of churning and, in turn, better maximize SNAP’s impact on nutrition security and health.

## Notes

Supplement. **eAppendix.** Explanation of Recertification Procedures **eFigure.** Flow Chart of Observations Included in Primary Analyses **eTable 1.** Characteristics of Recertification Forms Submitted by SNAP Households With Young Children Overall and by Outcome, Using 120 Day Definition Of Churn, May-August 2019 (N=5838 Households) **eTable 2.** Association of Race/Ethnicity and Demographic Factors With the Probability of Experiencing a Churn Spell at Annual Recertifications Within 120 Days of Certification Among Households With Young Children in Massachusetts, May-August 2019 (N=5838 Households) **eTable 3.** Changes by Sociodemographic Group in the Level and Trend of the Probability of Churning Associated With Administrative Policy Changes
