# Supplementary material for: Simplification of Supplemental Nutrition Assistance Program Recertification Processes and Association With Uninterrupted Access to Benefits Among Participants With Young Children
Source: JAMA Netw Open. 2022 Sep 6;5(9):e2230150. doi: 10.1001/jamanetworkopen.2022.30150 (PMC9449793; doi:10.1001/jamanetworkopen.2022.30150)
Supplement: Supplement. — eAppendix. Explanation of Recertification Procedures eFigure. Flow Chart of Observations Included in Primary Analyses eTable 1. Characteristics of Recertification Forms Submitted by SNAP Households With Young Children Overall and by Outcome, Using 120 Day Definition Of Churn, May-August 2019 (N=5838 Households) eTable 2. Association of Race/Ethnicity and Demographic Factors With the Probability of Experiencing a Churn Spell at Annual Recertifications Within 120 Days of Certification Among Households With Young Children in Massachusetts, May-August 2019 (N=5838 Households) eTable 3. Changes by Sociodemographic Group in the Level and Trend of the Probability of Churning Associated With Administrative Policy Changes [file jamanetwopen-e2230150-s001.pdf]

Supplementary Online Content

Kenney EL, Soto MJ, Fubini M, Carleton A, Lee M, Bleich SN. Simplification of Supplemental Nutrition Assistance Program recertification processes and association with uninterrupted access to benefits among participants with young children. *JAMA Netw Open*. 2022;5(9):e2230150. doi:10.1001/jamanetworkopen.2022.30150

eAppendix. Explanation of Recertification Procedures

eFigure. Flow Chart of Observations Included in Primary Analyses

eTable 1. Characteristics of Recertification Forms Submitted by SNAP Households With Young Children Overall and by Outcome, Using 120 Day Definition Of Churn, May-August 2019 (N=5838 Households)

eTable 2. Association of Race/Ethnicity and Demographic Factors With the Probability of Experiencing a Churn Spell at Annual Recertifications Within 120 Days of Certification Among Households With Young Children in Massachusetts, May-August 2019 (N=5838 Households)

eTable 3. Changes by Sociodemographic Group in the Level and Trend of the Probability of Churning Associated With Administrative Policy Changes

This supplementary material has been provided by the authors to give readers additional information about their work.

eAppendix. Explanation of Recertification Procedures

A client can apply for SNAP benefit recertification by applying online, submitting an application by mail or fax, dropping off an application, or meeting with a case manager in person.

Once an application is filed, a subsequent interview with the applicant is scheduled to ask him/her to provide any outstanding verification(s) that is needed to make an eligibility determination. Income, assets, and expenditures must all be verified before eligibility can be approved. Eligibility determinations are made within 30 days of the date the application is filed.

At recertification, when a case is closed, and the client returns to DTA within 30 days of the action, staff must determine if there was a delay in processing and whether the fault is attributed to the Department or to the client.

If the Department caused the delay, and the client is otherwise eligible, SNAP benefits must be approved retroactively to the date of application or to the day after the closing date.

If the client caused the delay, and is otherwise eligible, SNAP benefits must be prorated based on the remaining days of the month.

© 2022 Kenney EL et al. *JAMA Network Open.*

eFigure. Flow Chart of Observations Included in Primary Analyses

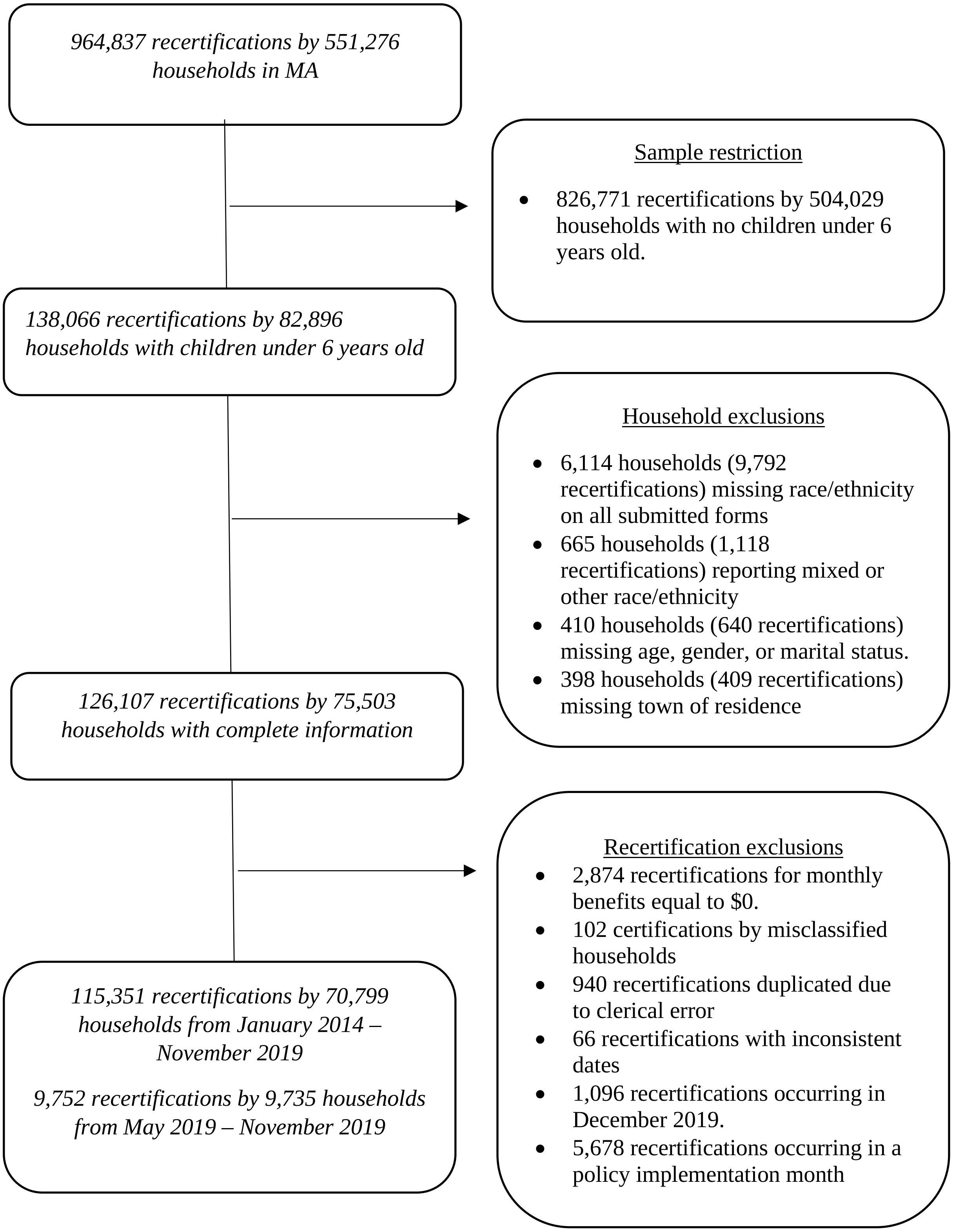

© 2022 Kenney EL et al. *JAMA Network Open.*

eTable 1. Characteristics of Recertification Forms Submitted by SNAP Households With Young Children Overall and by Outcome, Using 120 Day Definition Of Churn, May-August 2019 (N=5838 Households)

	Overall	Successful re-certification	Churn <30 days	No return
**Overall**	5,838	1,726 (29.6%)	2,903 (49.7%)	1,209 (20.7%)
Household Size, mean (SD)	4.21 (1.29)	4.34 (1.32)	4.23 (1.28)	3.99 (1.24)
Earned income, mean (SD)	853.64 (1023.78)	783.20 (1007.36)	774.89 (951.81)	1143.27 (1154.59)
Unearned income, mean (SD)	221.93 (440.95)	253.06 (469.04)	209.49 (422.36)	207.34 (441.39)
Monthly SNAP benefit, mean (SD)	399.70 (192.88)	418.23 (195.09)	419.29 (186.29)	326.22 (187.68)
Length of churn spell, mean (SD)			3.05 (5.33)	
**Race/Ethnicity**				
White, NH	2,123 (36.4%)	636 (36.8%)	1,014 (34.9%)	473 (39.1%)
Black, NH	1,328 (22.7%)	380 (22.0%)	660 (22.7%)	288 (23.8%)
Hispanic	2,257 (38.7%)	682 (39.5%)	1,167 (40.2%)	408 (33.7%)
Asian, NH	130 (2.2%)	28 (1.6%)	62 (2.1%)	40 (3.3%)
**Age group**				
17-29	2,703 (46.3%)	781 (45.2%)	1,376 (47.4%)	546 (45.2%)
30-39	2,473 (42.4%)	771 (44.7%)	1,192 (41.1%)	510 (42.2%)
40+	662 (11.3%)	174 (10.1%)	335 (11.5%)	153 (12.7%)
**Number of children <6 in household**				
1	4,374 (74.9%)	1,277 (74.0%)	2,119 (73.0%)	978 (80.9%)
2+	1,464 (25.1%)	449 (26.0%)	784 (27.0%)	231 (19.1%)
**Gender**				
Female	5,479 (93.9%)	1,649 (95.5%)	2,738 (94.3%)	1,092 (90.3%)
Male	359 (6.1%)	77 (4.5%)	165 (5.7%)	117 (9.7%)

© 2022 Kenney EL et al. *JAMA Network Open.*

eTable 2. Association of Race/Ethnicity and Demographic Factors With the Probability of Experiencing a Churn Spell at Annual Recertifications Within 120 Days of Certification Among Households With Young Children in Massachusetts, May-August 2019 (N=5838 Households)

	Estimate (95% CI)[b]	P-value
**Age group**		
18-29 (Reference group)	49.7 (47.6, 51.7)	<.001
30-39	-0.7 (-3.2, 1.7)	0.55
40+	3.3 (-0.9, 7.5)	0.12
**Race/Ethnicity**		
White, NH (Reference group)	48.0 (46.0, 50.0)	<.001
Black, NH	1.7 (-1.0, 4.4)	0.23
Hispanic	3.4 (0.7, 6.1)	0.02
Asian, NH	1.3 (-7.3, 10.0)	0.76
**Number of children <6 years old**		
1 (Reference group)	48.3 (46.7, 49.9)	<.001
2+	5.6 (2.7, 8.6)	<.001
**Gender**		
Female (Reference group)	49.9 (48.6, 51.2)	<.001
Male	-2.5 (-8.7, 3.7)	0.44
**Total household income**		
Per $1000 change (Mean $1100)	-4.2 (-5.3, -3.0)	<.001

© 2022 Kenney EL et al. *JAMA Network Open.*

eTable 3. Changes by Sociodemographic Group in the Level and Trend of the Probability of Churning Associated With Administrative Policy Changes

	Baseline Jan 2014 - Oct 2014		Policy 1 – Case banking Nov 2014 - Nov 2015		Policy 2 - Simplified reporting Dec 2015 - Apr 2019		Policy 3 - Virtual certification May 2019 - Dec 2019	
	Level	Trend	Level change	Trend change	Level change	Trend change	Level change	Trend change
**Age group**								
18-29 (Reference group)	**42.40 (40.54, 44.25)**	0.22 (-0.15, 0.59)	2.83 (-1.59, 7.25)	0.27 (-0.31, 0.85)	**-0.51 (-0.76, -0.27)**	**-0.51 (-0.76, -0.27)**	0.16 (-0.51, 0.84)	0.16 (-0.51, 0.84)
30-39	2.76 (-0.58, 6.11)	0.38 (-0.34, 1.11)	2.44 (-2.86, 7.74)	-0.62 (-1.34, 0.11)	0.12 (-0.22, 0.45)	0.12 (-0.22, 0.45)	-0.40 (-1.42, 0.62)	-0.40 (-1.42, 0.62)
40+	2.88 (-2.94, 8.70)	0.67 (-0.42, 1.76)	-0.85 (-8.65, 6.96)	-0.68 (-1.85, 0.49)	-0.07 (-0.70, 0.55)	-0.07 (-0.70, 0.55)	-0.46 (-1.98, 1.05)	-0.46 (-1.98, 1.05)
**Race/Ethnicity**								
White, NH (Reference group)	**43.21 (41.57, 44.86)**	0.18 (-0.20, 0.56)	2.73 (-0.94, 6.41)	-0.22 (-0.73, 0.30)	**-0.46 (-0.75, -0.16)**	**-0.46 (-0.75, -0.16)**	-0.26 (-1.14, 0.62)	-0.26 (-1.14, 0.62)
Black, NH	4.03 (-0.93, 8.99)	-0.55 (-1.30, 0.20)	2.61 (-3.53, 8.75)	0.50 (-0.32, 1.33)	0.04 (-0.61, 0.69)	0.04 (-0.61, 0.69)	0.16 (-1.38, 1.69)	0.16 (-1.38, 1.69)
Hispanic	**5.47 (2.55, 8.38)**	-0.20 (-0.79, 0.38)	1.37 (-2.83, 5.58)	0.18 (-0.39, 0.74)	-0.03 (-0.36, 0.31)	-0.03 (-0.36, 0.31)	0.23 (-0.99, 1.45)	0.23 (-0.99, 1.45)
Asian, NH	5.26 (-3.31, 13.83)	-0.15 (-1.47, 1.17)	-2.89 (-10.70, 4.91)	0.31 (-1.52, 2.14)	-0.30 (-1.24, 0.64)	-0.30 (-1.24, 0.64)	0.43 (-2.54, 3.40)	0.43 (-2.54, 3.40)
**Number of kids <6**								
1 (Reference group)	**42.65 (40.85, 44.44)**	0.20 (-0.16, 0.56)	2.65 (-0.56, 5.85)	-0.09 (-0.48, 0.29)	**-0.54 (-0.74, -0.33)**	**-0.54 (-0.74, -0.33)**	0.07 (-0.50, 0.64)	0.07 (-0.50, 0.64)
2+	2.95 (-0.44, 6.34)	-0.40 (-1.05, 0.25)	4.47 (-0.39, 9.34)	0.21 (-0.57, 0.98)	0.22 (-0.20, 0.64)	0.22 (-0.20, 0.64)	-0.77 (-1.99, 0.45)	-0.77 (-1.99, 0.45)
**Gender**								
Female (Reference group)	**42.71 (40.95, 44.48)**	0.19 (-0.17, 0.56)	**3.49 (0.66, 6.32)**	-0.01 (-0.38, 0.36)	**-0.49 (-0.67, -0.32)**	**-0.49 (-0.67, -0.32)**	-0.11 (-0.58, 0.36)	-0.11 (-0.58, 0.36)
Male	-4.42 (-10.22, 1.37)	0.19 (-0.78, 1.17)	3.46 (-3.01, 9.93)	-0.38 (-1.54, 0.79)	0.16 (-0.49, 0.80)	0.16 (-0.49, 0.80)	-0.02 (-1.97, 1.93)	-0.02 (-1.97, 1.93)

Source: Authors' analysis of data from Massachusetts Department of Transitional Assistance and US Bureau of Labor Statistics.
Note: Sample includes SNAP participant head of households with at least one child less than 6 years old. Robust standard errors were clustered on participant's town of residence. Models include indicators for calendar month and monthly unemployment rates for town of residence. Bold indicates statistically significant difference from zero for the reference groups or a statistically significant difference from the reference group for each sub-group at p<.05
"Casebanking" allowed SNAP participants to conduct their recertification application and interview with the first available caseworker instead of caseworkers being assigned at the beginning of the certification period.
"Simplified reporting" reduced the requirements for reporting changes in income or household composition to be once a year rather than having to report them as they occurred.
"Virtual certification" permitted SNAP participants to submit recertification requirements to an online portal rather than in-person.

© 2022 Kenney EL et al. *JAMA Network Open.*
